# The Volatile Flavor Substances, Microbial Diversity, and Their Potential Correlations of Inner and Surface Areas within Chinese Qingcheng Mountain Traditional Bacon

**DOI:** 10.3390/foods12203729

**Published:** 2023-10-11

**Authors:** Hongfan Chen, Yulin Zhang, Xinyi Wang, Xin Nie, Dayu Liu, Zhiping Zhao

**Affiliations:** 1 Meat Processing Key Laboratory of Sichuan Province, Chengdu University, Chengdu 610106, Chinaliudayu@cdu.edu.cn (D.L.); 2 College of Food Science and Technology, Sichuan Tourism University, Chengdu 610100, China; 3 School of Basic Medical Sciences, Chengdu Medical College, Chengdu 610500, China

**Keywords:** Chinese traditional bacon, microbial community structure, key flavor substance, multivariate statistical analysis, correlation analysis

## Abstract

The objective of this study was to explore the microbial diversity, volatile flavor substances, and their potential correlations in inner and surface Chinese Qingcheng Mountain traditional bacon (CQTB). The results showed that there were 39 volatile flavor substances in inner and surface CQTB detected by headspace solid-phase microextraction and gas chromatography–mass spectrometry (HS-SPME-GC-MS). Moreover, significant differences in volatile flavor substances between the inner and surface CQTB were observed. Sixteen key volatile flavor substances were screened (OAV > 1), including guaiacol, nonanal, ethyl isovalerate, and others. High-throughput sequencing (HTS) result indicated that Firmicutes, Proteobacteria, and Actinobacteria were the predominant bacterial phyla, and Ascomycota and Mucoromycota were the predominant fungal phyla. *Staphylococcus*, *Psychrobacter*, and *Brochothrix* were the predominant bacteria, and *Debaryomyces*, *Penicillium*, and *Mucor* were the predominant fungal genera. Spearman correlation coefficient analysis suggested that *Apiotrichum* and *Lactobacillus* were closely and positively correlated with the formation of key phenol compounds. The present work demonstrates the microbial diversity and related volatile flavor substances and their potential correlations in CQTB and provides a theoretical basis for the development of microbial starter culture and green processing of CQTB.

## 1. Introduction

Bacon is one of the most representative traditional meat products in China [1]. It is a non-ready-to-eat meat product with livestock meat as raw material processed through a series of procedures such as curing, air-drying, smoking, or non-smoking after the addition of auxiliary materials [2]. Sichuan bacon is widely favored by consumers in China because of its unique flavor, bright color, and rich aroma [3]. According to incomplete statistics, the consumption of cured meat products accounts for approximately 60% of the total meat products in Sichuan province, among which bacon accounts for a higher proportion. 

Flavor is normally considered one of the most important qualities of meat products. In recent years, quantitative analyses of volatile flavor substances in smoked bacon have been extensively reported [4]. Phenols, aldehydes, ketones, and acids are demonstrated as the main volatile flavor substances in smoked bacon. Xi et al. (2021) identified 40 volatile flavor substances in Chinese Zhenba bacon by gas chromatography–mass spectrometry (GC−MS), including ketones, phenols, alcohols, esters, and aldehydes [5]. Thirty-two key aroma compounds were screened in bacon from different areas of China based on odor activity values, including heptanal, nonanal, 1-octen-3-ol, and (E)-2-nonenal [6]. The smoked bacon flavor is closely related to processing conditions, including curing and smoking. Another study showed that ultrasonic-assisted curing significantly enhanced the levels of ester compounds in bacon [7]. On the other hand, smoke flavor is the major characteristic flavor distinguishing smoked bacon from other meat products [8]. Yang et al. (2023) found that smoking altered bacon flavor characteristics and significantly increased the abundance of flavor-related microorganisms [9].

Environmental microorganisms often greatly influence the quality of traditional meat products [10]. *Staphylococcus*, *Macrococcus*, *Debaryomyces*, and *Candida* were the dominant microorganisms in the meat ecosystem [11]. Lipase, proteases, and catalases produced by functional microorganisms promote the hydrolysis of fats and the degradation of proteins in the meat to produce fatty acids, peptides, free amino acids, and volatile organic compounds, which form the flavor compounds of Chinese bacon [3,12]. Song et al. (2022) suggested that *Staphylococcus* (*S.*) *equorum* was the dominant bacteria in Sichuan traditional bacon, and the dominant fungi were *Candida zeylanoides* and *Debaryomyces prosopidis* [2]. At the genus level, *Aspergillus*, *Candida*, *Debaryomyces*, *Malassezia*, and *Penicillium* were considered the dominant fungus in Dazhou bacon, Sichuan province [13]. *Staphylococcus* was the predominant bacteria in the bacon produced in eight different cities in Sichuan province [14]. On the other hand, *S. xylose*, *Lactobacillus* (L.) *plantarum*, and *Leuconostoc mesenteroides* can significantly increase the types and contents of volatile flavor substances in Chinese bacon [15]. Moreover, *Staphylococcus* and *Salinivibrio* are closely related to regulate and shape the Sichuan—Chongqing bacon flavor substances [9].

Qingcheng Mountain is a suitable place for producing traditional Chinese bacon, with an average temperature ranging from 7 °C to 9 °C and a relative humidity of approximately 80%. The Chinese Qingcheng Mountain traditional bacon (CQTB) is very popular because of its bright red color, rich smoky taste, hard texture, and good chewing taste. However, the CQTB is still produced with traditional technologies and spontaneous fermentation. Therefore, the production of the CQTB normally depends on skills and experiences rather than a process totally based on modern scientific technology. Theoretically, the differences in microbial diversity and volatile flavor substances between the inner and surface CQTB final product are significant. At present, however, literature on the volatile flavor substances and microbial diversity of the inner and surface CQTB final product has not yet been reported. Moreover, potential correlations between microbial diversity and volatile flavor substances in the inner and surface CQTB final product are still unknown. In this study, the volatile flavor substances of the inner and surface CQTB final product were investigated by GC-MS coupled with E-nose. Moreover, the microbial diversity of the inner and surface CQTB final product was analyzed by high-throughput sequencing (HTS). Finally, the potential correlations among the dominant microflora, volatile flavor substances, and physicochemical indicators were proposed based on Spearman correlation coefficient analysis. The experimental results supply a theoretical basis for the development of microbial starter culture and green processing of the CQTB.

## 2. Materials and Methods

### 2.1. Sample Preparation

The CQTB was prepared in Zhao’s Old Bacon in Qingcheng Mountain, Dujiangyan City, Sichuan Province, China. When preparing the CQTB, the pork belly from one pig was divided into strips (length 40–45 cm, width 5–8 cm, and thickness 4–7 cm). The meat strips with sodium chloride (3.5%, *m*/*m*) were then cured for 24 h, followed by smoking by cypress wood at 50–55 °C for 36 h. The meat strips were taken out after smoking, cooled to room temperature (5–8 °C), then stored at room temperature (7–15 °C) for 2 months. Three batches of the bacons were prepared. After sampling, the samples were immediately transported to the laboratory in a sealed aseptic bag at a low temperature. The meat within a 1.5 cm distance from the surface was defined as the surface bacon, while the meat more than 1.5 cm distance from the surface was defined as the inner bacon. Three batches of the CQTB were sampled and mixed completely before analysis. The physicochemical properties were immediately measured. The rest of the samples were stored at −80 °C in vacuum-packaged aluminum foil bags for GC-MS, E-nose, and HTS analysis.

### 2.2. Physicochemical Analysis

The pH values were determined according to the method of Jin et al. (2010) [16] by using a pH meter (PHS-3C, Shanghai Sanxin Instrument, Shanghai, China). The water activity was determined according to Wang et al. (2018) [17]. Five grams of minced CQTB was measured in a water activity meter (HD-5, Wuxi Huake Instrument, Wuxi, China) at 25 °C. The moisture content was determined according to Guo et al. (2016) [18] by using a moisture analyzer (Guanya Moisture Analyzer, Shenzhen Guangya Technology, Shenzhen, China). The redness (a*), yellowness (b*), and brightness (L*) of the CQTB were measured using a colorimeter (RC-10, Konica Minolta, Tokyo, Japan). All experiments were repeated three times.

### 2.3. Determination of Volatile Flavor Substances by GC-MS

The volatile flavor substances were detected using an Agilent 7890B gas chromatograph and an Agilent Model 5977 MSD series mass selective detector with a quadrupole mass analyzer (Agilent Technologies, Inc., Santa Clara, CA, USA) [19]. Three grams of the CQTB was filled in a headspace flask (15 mL). The pretreatment conditions for the CQTB samples with an automatic injector (CTC Analytics AG, Zwingen, Switzerland) were performed according to our previous study [20]. For qualitative analysis, the flavor data were retrieved and matched in the NIST14.L library of the instrument, and the substances with a matching degree of more than 80% were selected. The experiments were performed three times for every sample.

### 2.4. E-Nose Detection

A Fox 4000 Sensory Array Fingerprint Analyzer (Alpha M.O.S., Toulouse, France) was employed for E-nose. A half gram of minced CQTB was accurately weighed, filled into a headspace bottle (15 mL), and balanced for 5 min at 70 °C. The detection and analysis were performed according to our previous study [20]. A principal component analysis (PCA) plot of the E-nose was carried out for further analysis. The experiments were performed three times for every sample.

### 2.5. DNA Extraction, PCR Amplification, and Sequencing

DNA extraction from the CQTB and amplifications of V3–V4 and ITS1–ITS2 regions as well as the sequencing were performed as described in a previous study [20]. Three parallel experiments were performed for the inner and surface CQTB.

### 2.6. Data Processing

Data processing was performed using IBM SPSS version 24 (IBM, Inc., Armonk, NY, USA). The PCA and orthogonal partial least squares discrimination analysis (OPLS-DA) were conducted by SIMCA 14.1 software (Umetrics, Umea, Sweden). A one-way analysis of variance (ANOVA) was performed among the means using Tukey’s honest significant difference (HSD) to analyze the differences between the inner and surface CQTB (*p* < 0.05). The results were expressed as mean values ± standard errors. The correlations among the dominant microorganisms, volatile flavor substances, and physicochemical indicators were calculated by Spearman correlation coefficients. The cluster dendrogram, alpha diversity box plot, chord diagram, and correlation heatmaps were visualized through R 4.2.3. The radar map of the E-nose was drawn using Origin 2021 software (OriginLab, Northampton, MA, USA).

## 3. Results and Discussion

### 3.1. Physicochemical Properties Analysis

The significant difference in pH value, water activity, moisture content, b*, and L* was not observed between the inner and surface CQTB, as shown in Table 1. However, the a* of the surface CQTB was dramatically higher than that of the inner CQTB (*p* < 0.05). It has been well demonstrated that smoking will increase the bacon’s redness [21]. On the other hand, the a* value of the CQTB is closely related to lipid oxidation and myoglobin degradation. Since the surface CQTB was exposed to air much stronger than the inner CQTB, the red color gradually decreased from 7.380 to 5.390, resulting in lower redness in the surface CQTB.

### 3.2. Volatile Flavor Substances in CQTB

**Figure 1:**
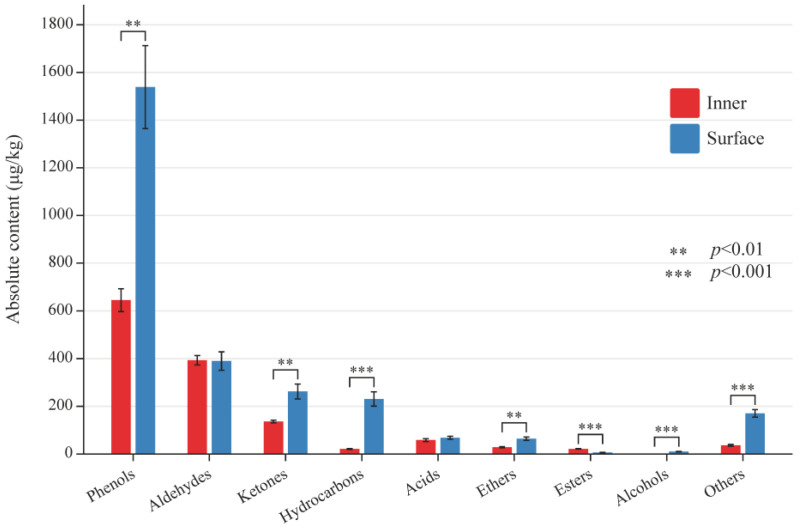
Absolute content of volatile compounds in the inner and surface CQTB.

It has been well documented that flavors of meat products are produced mainly through lipid and protein degradation, Maillard reactions, and Strecker degradation [22]. The levels of volatile flavor substances ranged from 0.82 μg/kg to 310.71 μg/kg. Thirty-nine volatile flavor substances were identified in the inner and surface CQTB (Table S1). The absolute content of volatile compounds in the inner and surface CQTB was shown in Figure 1. There were 29 and 35 volatile flavor substances in the inner and surface CQTB, respectively. Furthermore, 25 compounds were identified in both the inner and surface CQTB, including 10 phenols, 4 ketones, 3 alkanes, 2 aldehydes, 1 ester, 1 acid, 1 ether, and 3 others. The characteristic substances in the inner CQTB included n-hexanal, ethyl 2-methyl butyrate, octanoic acid, and n-hexadecane. However, the characteristic substances in the surface CQTB were 3-methyl-2-cyclopenten-1-one, 2-ethyl-2-hexenal, 1-octene-3-ol, phenylacetaldehyde, dodecane, 6-(acetoxy)-4-methyl-4-hexenal, 2-nonanone, 2,5-dimethylphenol, 3-ethyl-5-methylphenol, and 2-methylnaphthalene.

Aldehydes are the major contributors to the overall flavor of meat products with low odor threshold [23]. Because of the low threshold and strong volatility, aldehydes are usually considered important volatile flavor substances in cured meat products [24]. Heptanal and nonanal were detected in both the inner and surface CQTB, probably originating from oxidations of linoleic acid and some other unsaturated fatty acid [25,26]. N-hexanal was only detected in the inner CQTB, which might be due to the difference in the oxidation degree of the inner and surface CQTB. In addition, n-hexanal gave a green and fruity aroma to foods at a low concentration. However, n-hexanal brought grass flavor when its concentration reached 4.5 μg/kg, which, therefore, negatively affected the flavor of food [27]. It has previously been shown that n-hexanal gives a fruity and broth-like odor at a low concentration, while it has a rancid odor at high concentrations [28]. Nonanal normally gives a greasy and sweet orange flavor [29]. The concentration of nonanal detected in the surface of CQTB was significantly higher than in the inner CQTB. It is well known that the formation of nonanal is closely related to woodchip types [21]. The effects of smoke on the surface CQTB were stronger than on the inner CQTB. Therefore, the concentration of nonanal in the surface CQTB was more than that in the inner CQTB. Phenylacetaldehyde is a catabolism product of phenylalanine and was only detected in the surface CQTB [30], giving the bacon a honey flavor.

Ketones are mainly generated from the degradation of amino acids, oxidation of unsaturated fatty acids and β-keto acid, and carbohydrate metabolism [31], which normally represent fruity, woody aroma, and mushroom-like flavor [32]. 3-Methyl-2-cyclopenten-1-one, the typical volatile compound in smoked sausage and bacon [21,33], was only detected in the surface CQTB. It has been reported that the level of 3-methyl-2-cyclopenten-1-one is related to the woodchip types [34]. Compared with aldehydes, ketones have a higher threshold and modify the flavor of the CQTB. The key ketone in this study was 2-nonanone, which derived from β-lipid oxidation [35], giving the CQTB grass flavor.

Acids are usually produced from lipid autoxidation and aldehyde oxidation [36]. In this work, two acids in the CQTB were identified. Octanoic acid was only detected in the inner CQTB. It has been suggested that the generation of octanoic acid is normally under non-smoking conditions [33]. Therefore, it was reasonable that the octanoic acid was only detected in the inner CQTB.

Phenols originated from lignin pyrolysis and greatly contributed to the flavor of smoked meat products [37]. On the other hand, during the microbial enzymatic reaction and wood combustion, lignin and phenolic acids were decomposed to produce phenol compounds, which gave bacon a woody, spicy, and smoked flavor [38]. In this study, 10 and 12 phenols were detected in the inner and surface CQTB, respectively. Guaiacol, the most important phenol substance in smoked meat products, and its homologues produced during smoking were the main sources of the smoking flavor [39,40]. In the present study, the absolute content of guaiacol in the inner and surface CQTB was 161.04 μg/kg and 244.33 μg/kg, respectively, with an olfactory threshold of 1.6 μg/kg (Table 2). Varlet et al. (2006) suggested that guaiacol was the phenol compound in smoked salmon, which was responsible for the smoked odor [41]. 2,6-Dimethoxyphenol, 4-ethyl-2-methoxyphenol, 2-methoxy-5-methylphenol, o-cresol, and p-cresol were also considered key volatile phenols in the present study.

Because of the high olfactory threshold value, hydrocarbons have relatively less influence on bacon flavor formation [42]. In this study, 6 hydrocarbons were identified in the CQTB, including dodecane, n-octane, and n-hexadecane (Table S1). Dodecane was only detected in the surface CQTB, while n-hexadecane was only detected in the inner CQTB. The n-octane was identified in both the inner and surface CQTB. However, the content of n-octane in the surface CQTB was significantly higher than that in the inner CQTB.

### 3.3. Multivariate Statistical Analysis of Volatile Flavor Substances in CQTB

**Figure 2:**
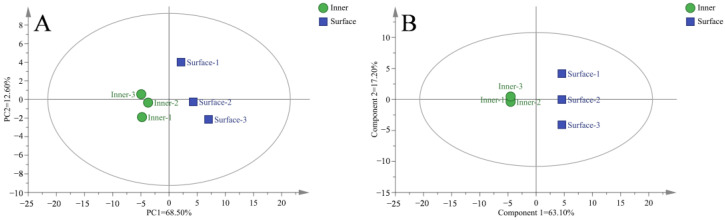
The PCA model (**A**) and OPLS-DA (**B**) scatter plots for the inner and surface CQTB.

The multivariate statistical analysis for volatile flavor substances of the CQTB was performed using the standardized operation method of automatic proper calculation (Unit Variance Scaling, UV). Figure 2A,B show the PCA and OPLS-DA scatter plots of the inner and surface CQTB, respectively. Obviously, the inner and surface CQTB was well placed in different regions, suggesting significant differences in volatile flavor substances between the inner and surface CQTB. The parameters for the PCA model were R2X = 0.856 and Q2 = 0.590, while the parameters for the OPLS-DA model were R2X = 0.957 and Q2 = 0.999. The results suggested that the model could well explain the differences in volatile flavor substances between the inner and surface CQTB.

### 3.4. Key Flavor Substances in CQTB

The concentration and threshold of volatile flavor substances are key factors affecting the overall flavor of meat products [43]. Sixteen key volatile flavor substances were identified based on their odor activity value (OAV > 1) using the relevant references [44,45], as listed in Table 2, including 7 phenols, 4 aldehydes, 2 esters, 1 alcohol, 1 ketones, and 1 other. It is worth mentioning that these 16 volatile flavor substances all showed significant differences in the OAV between the inner and surface bacon (*p* < 0.05; Table 2). The OAVs for 1-nonanal, 2-methoxy-5-methylphenol, 4-ethyl-2-methoxyphenol, ethyl isovalerate, eugenol, guaiacol, heptanal, and p-cresol in both the inner and surface CQTB were greater than 1, which shows that these substances are among the most important flavor compounds in smoked, cured meat. Ethyl isovalerate has a fruity aroma, while guaiacol has vanilla and woody aromas, making it an important source of the characteristic flavor of smoked bacon. Moreover, 2-methylnaphthalene, 2-nonanone, 1-octene-3-ol, 4-ethylphenol, 2-methoxy-4-propylphenol, and phenylacetaldehyde were the key flavor compounds in the surface CQTB, providing woody, grassy, smoked, and clove aromas for the CQTB. Meanwhile, ethyl 2-methylbutyrate and hexanal provided fruit and smoked flavors being key flavor components in the inner CQTB.

**Table 2:** Key volatile flavor substances in CQTB.

Compound	CAS	OT (µg/kg)	OAV	
			Inner	Surface
1-Nonanal	124-19-6	0.008	21,102.54 ± 1531.25 ^b^	28,465.82 ± 1019.91 ^a^
2-Methoxy-5-methylphenol	1195-09-1	13	9.23 ± 1.54 ^b^	18.97 ± 1.63 ^a^
2-Methylnaphthalene	91-57-6	3	-	4.21 ± 0.42 ^a^
2-Nonanone	821-55-6	41	-	1.36 ± 0.31 ^a^
1-Octene-3-ol	3391-86-4	0.0015	-	6546.66 ± 800.00 ^a^
4-Ethyl-2-methoxyphenol	2785-89-9	16	3.88 ± 0.32 ^b^	10.11 ± 1.74 ^a^
4-Ethylphenol	123-07-9	21	0.62 ± 0.03 ^b^	2.1 ± 0.24 ^a^
2-Methoxy-4-propylphenol	2785-87-7	10	0.25 ± 0.05 ^b^	2.65 ± 0.41 ^a^
Ethyl 2-methylbutyrate	7452-79-1	0.063	52.62 ± 5.98 ^a^	-
Ethyl isovalerate	108-64-5	0.11	163.20 ± 40.05 ^a^	57.13 ± 5.55 ^b^
Eugenol	97-53-0	2.5	1.11 ± 0.10 ^b^	4.52 ± 0.51 ^a^
Guaiacol	90-05-1	1.6	100.63 ± 12.54 ^b^	152.71 ± 12.07 ^a^
Heptanal	111-71-7	2.8	14.48 ± 4.44 ^a^	11.09 ± 1.44 ^b^
Hexanal	66-25-1	4.5	40.91 ± 9.34 ^a^	-
P-cresol	106-44-5	10	9.21 ± 0.85 ^b^	31.07 ± 3.07 ^a^
Phenylacetaldehyde	122-78-1	0.004	-	12,739.84 ± 2754.70 ^a^

Different superscript letters in the same row suggest significant differences (*p* < 0.05); CAS: Chemical Abstract Services registry number; OT: olfactory threshold value; OAV: odor activity value [45].

### 3.5. E-Nose Analysis of CQTB

**Figure 3:**
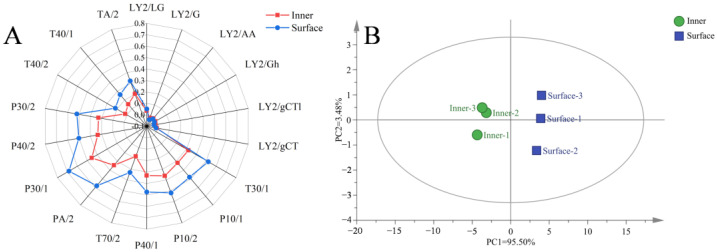
E-nose analysis for the inner and surface CQTB. (**A**,**B**) respectively represent radar chart and principal component analysis.

Different E-nose sensors represent their specific characteristics, as shown in Figure 3A. The inner and surface CQTB was more sensitive to P30/1, P30/2, PA/2, P40/1, P10/1, and T30/1 sensors, which were sensitive to organic compounds, hydrocarbons, amines, ethers, and alcohols [46]. The PA/2 sensor indicating hydrocarbons had the biggest difference between the inner and surface CQTB. It agreed well with the GC-MS results that 1-octene-3-ol and phenylacetaldehyde were only identified as key volatile flavor substances in the surface bacon.

The overall volatile flavors of the inner and surface CQTB were analyzed by E-nose. The PCA was employed to visualize samples’ attributes and variable relationships between the inner and surface CQTB, as revealed in Figure 3B. The total contribution rate of PC1 (95.50%) and PC2 (3.48%) was 98.98%, which accurately represented the overall results of the E-nose. It was apparent that the inner and surface CQTB had significant differences since the inner CQTB was placed at the left of the coordinate axis, while the surface CQTB was placed at the right of the coordinate axis.

### 3.6. Analysis of Microbial Diversity in CQTB

**Figure 4:**
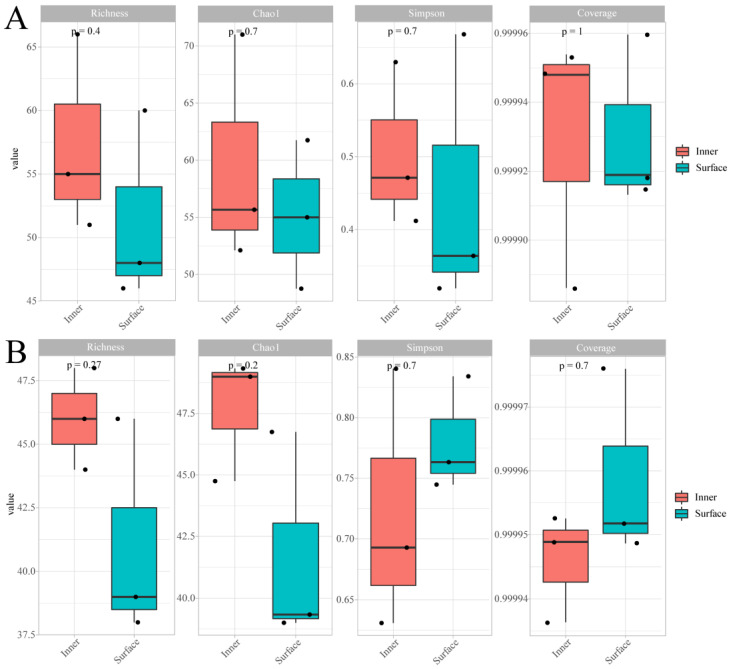
Alpha diversity of bacteria (**A**) and fungus (**B**) in inner and surface CQTB.

Figure 4 shows the α-diversity of bacteria and fungi in the CQTB. The Richness and Chao1 values of bacteria and fungus in the inner CQTB were higher than those in the surface CQTB. The bacteria Simpson index value of the surface CQTB was lower than that in the inner CQTB. However, the fungus Simpson index value in the inner CQTB was lower than that in the surface CQTB. On the other hand, the bacteria Richness and Chao1 value were higher than those of the fungus, and the bacteria Simpson index value was lower than that of the fungus, indicating that the richness and diversity of bacteria were higher than those of the fungus. The Coverage suggested that the sequencing depth was enough to reflect the information on bacterial and fungal species in the CQTB. Moreover, the bacterial microbial taxa in the CQTB were more abundant than that of the fungal microbial taxa.

**Figure 5:**
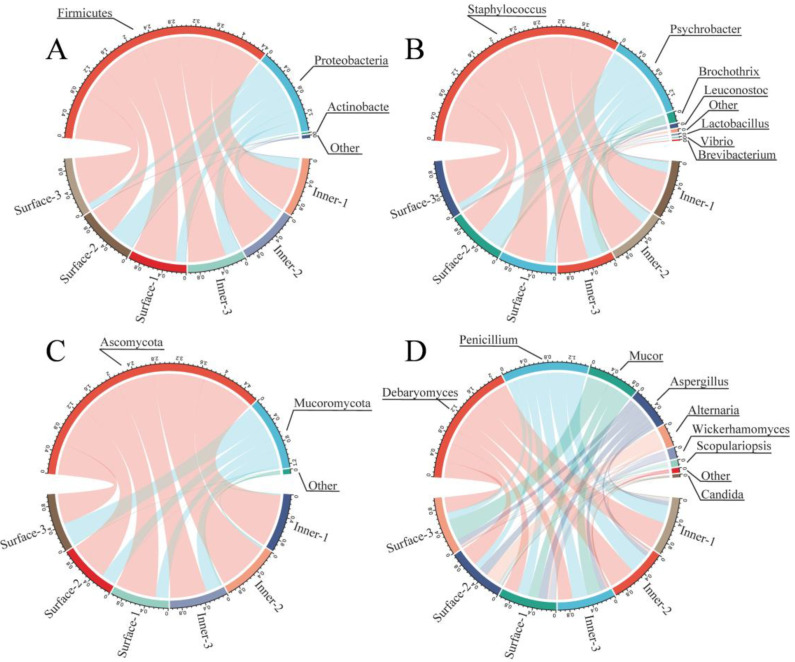
Relative abundance of bacteria and fungi at the phylum and genus levels in the inner and surface CQTB. (**A**,**B**) respectively represent bacteria phylum and genus; (**C**,**D**) respectively indicate fungi phylum and genus.

Based on 16S rRNA sequencing, 8 phyla and 41 genera in CQTB were identified. Bacteria with a relative abundance of more than 0.1% were considered the dominant bacteria, as revealed in Figure 5. At the phylum level, Firmicutes (inner 75.67%, surface 73.15%), Proteobacteria (inner 23.30%, surface 26.11%), and Actinobacteria (inner 0.27%, surface 0.19%) were the identified dominant bacteria, which was consistent with Song et al. (2022) on the Sichuan smoked bacon [2] and Wang et al. (2022) on the Chinese traditional sausage [3]. Furthermore, significant difference was not observed in the bacterial phylum between the inner and surface CQTB. Seven dominant bacterial genera were demonstrated in the inner and surface CQTB, namely *Staphylococcus* (inner 70.21%, surface 70.40%), *Psychrobacter* (inner 23.27%, surface 25.68%), *Brochothrix* (inner 4.20%, surface 1.49%), *Leuconostoc* (inner 1.00%, surface 0.96%), *Lactobacillus* (inner 0.26%, surface 0.29%), *Brevibacterium* (inner 0.27%, surface 0.09%), and *Vibrio* (inner 0.03%, surface 0.43%). *Staphylococcus* was the most dominant bacteria in the CQTB, which agreed with Yang et al. (2022) [9]. *Staphylococcus* played an important role in improving the quality and safety of fermented meat through the inhibition of lipid oxidative decomposition [47]. *Staphylococcus* can secrete proteases, lipases, and nitrate reductases, which degrade the proteins, lipids, and other compounds into ketones, esters, acids, and other volatile flavor substances [48].

Based on HTS sequence analysis, 4 phyla and 16 genera were identified in the CQTB. Fungi with a relative abundance of more than 0.1% were considered the dominant fungus, as seen in Figure 5. Ascomycota (inner 87.05%, surface 68.52%) and Mucoromycota (inner 11.03%, surface 30.94%) were the dominant fungal phyla. In addition, Ascomycota accounted for the largest proportion, which was similar to Zhang et al. (2021) [13]. There were 8 dominant fungal genera in the CQTB, including *Debaryomyces* (inner 41.80%, surface 30.08%), *Penicillium* (inner 33.67%, surface 16.19%), *Mucor* (inner 9.24%, surface 20.04%), *Aspergillus* (inner 8.32%, surface 14.65%), *Alternaria* (inner 1.80%, surface 10.89%), *Scopulariopsis* (inner 1.63%, surface 1.76%), *Wickerhamomyces* (inner 0.97%, surface 5.04%), and *Candida* (inner 0.66%, surface 0.79%). *Debaryomyces* and *Penicillium* accounted for the largest proportions in the CQTB, in line with Song et al. (2022) [2] and Magistá et al. (2017) [49]. Mold plays an important role in meat products, as it is responsible for the development of the specific flavor of dried meat [50]. On the other hand, surface molds possess antioxidative effects and have a protective effect on pathogenic and spoilage microorganisms [43].

### 3.7. Correlation Analysis among Key Flavor Substances, Dominant Microflora, and Physicochemical Indicators of CQTB

**Figure 6:**
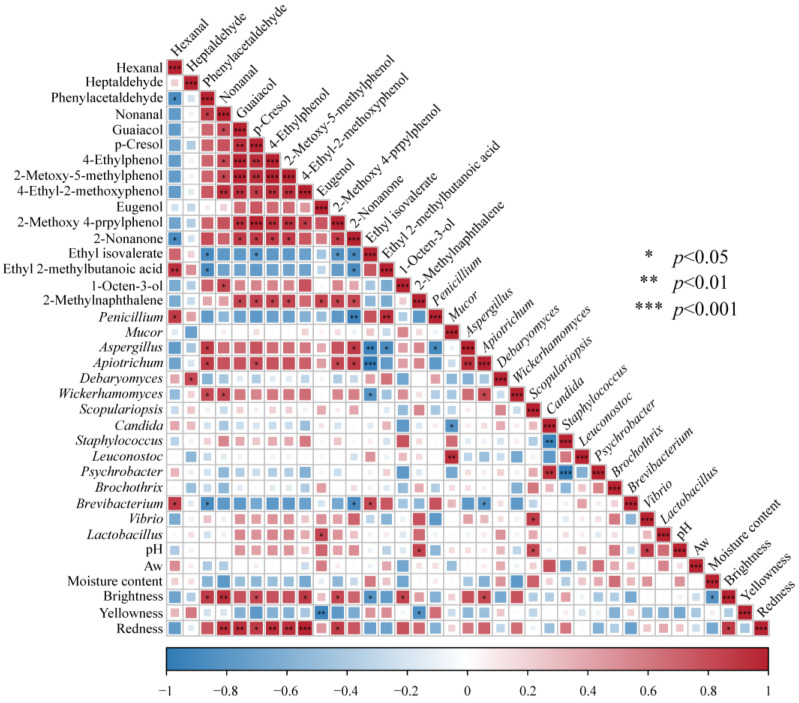
Heatmap of the correlations between key flavor substances, physicochemical indicators, and dominant microflora for CQTB.

Protein hydrolysis, lipid oxidation, and Maillard reaction greatly contribute to the flavor formation of meat products [51,52]. On the other hand, microorganisms also greatly contribute to the formation of flavor substances [53]. The potential correlations among key flavor substances, dominant microflora, and physicochemical indicators of the CQTB were established based on the calculation of the Spearman correlation coefficient, with the criteria of |r| ≥ 0.8 and *p* < 0.05, as shown in Figure 6. Yeast is considered the key fungi promoting meat product fermentation [54], contributing to the synthesis of aldehydes, esters, and other volatile flavor substances through glycolysis, protein degradation, and lipid oxidation. In the present study, phenylacetaldehyde was significantly positively correlated with *Wickerhamomyces* (r = 0.820, *p* < 0.05), which agreed well with Zhang et al. (2018) [55]. Similarly, heptanal had a positive correlation with *Debaryomyces* (r = 0.829, *p* < 0.05). All the key phenols had positive correlation with *Apiotrichum*. The p-cresol (r = 0.829, *p* < 0.05) and 2-methoxy-4-propylphenol (r = 0.829, *p* < 0.05) were most significantly positively correlated with *Apiotrichum*. Moreover, *Apiotrichum* was positively related to aldehydes and ketones formation, which was inconsistent with the previous study by Xu et al. (2022) [56]. This might be because the smoking process contributed to the growth of *Apiotrichum*, accompanying the formation of key phenols, resulting in a highly positive correlation between all key phenols and *Apiotrichum*. On the other hand, all the key phenols were also positively correlated with *Lactobacillus*. The eugenol was significantly positively correlated with *Lactobacillus* (r = 0.841, *p* < 0.05), which agreed with Jiang et al. (2022) [57]. Seven key phenols were positively correlated with redness and brightness, which was in line with Du et al. (2022) [21]. Furthermore, 2-nonanone had a positive correlation with *Aspergillus* (r = 0.880, *p* < 0.05) and *Apiotrichum* (r = 0.880, *p* < 0.05). Ethyl isovalerate was only positively correlated with *Brevibacterium* (r = 0.829, *p* < 0.05). However, ethyl isovalerate was negatively correlated with *Aspergillus* (r = −0.943, *p* < 0.01), *Apiotrichum* (r = −0.999, *p* < 0.001), and *Wickerhamomyces* (r = −0.829, *p* < 0.05). The correlation analysis showed that volatile flavor substances were closely related to microbial diversity, which affected the physicochemical properties of the CQTB and, thus, affected the product quality.

## 4. Conclusions

In this study, the inner and surface CQTB flavor and microbial substances, respectively, were analyzed by HS-SPME-GC-MS and HTS. A total of 39 volatile flavor substances were detected in the CQTB, which were mainly composed of phenols and aldehydes. Seventeen key volatile flavor substances with OAV > 1 were identified from the inner and surface CQTB. At the phylum level, Firmicutes, Proteobacteria, and Actinobacteria were the dominant bacteria, and Ascomycota and Mucoromycota were the dominant fungi. At the genus level, *Staphylococcus*, *Psychrobacter*, and *Brochothrix* were the dominant bacteria, and *Debaryomyces*, *Penicillium*, and *Mucor* were the dominant fungus. Correlation analysis showed that *Aspergillus*, *Apiotrichum*, and *Lactobacillus* possibly greatly contributed to the flavor formation of the CQTB. The present study just investigates the flavor and microbial diversity of the inner and surface of the final CQTB. Changes in microbial diversity and volatile flavor substances during the processing of the CQTB will be deeply investigated in future studies. Based on important roles of potential microorganisms in the quality of the CQTB, isolation, identification, and application of these functional microorganisms to promote the green processing of the CQTB will be performed in the future.

## Figures and Tables

**Table 1:** Comparison of physicochemical properties between the inner and surface CQTB.

	Inner	Surface
pH	5.710 ± 0.08 ^a^	5.680 ± 0.04 ^a^
Water activity	0.693 ± 0.02 ^a^	0.699 ± 0.01 ^a^
Moisture content (%)	8.740 ± 0.38 ^a^	9.040 ± 0.26 ^a^
Brightness (L*)	42.990 ± 1.69 ^a^	45.830 ± 0.79 ^a^
Redness (a*)	7.380 ± 0.36 ^a^	5.390 ± 0.29 ^b^
Yellowness (b*)	4.570 ± 0.31 ^a^	4.250 ± 0.30 ^a^

Different superscript letters in the same row indicate significant differences (*p* < 0.05).

## Data Availability

The data supporting the results of this study are included in the present article.

## Supplementary Materials

The following supporting information can be downloaded at: https://www.mdpi.com/article/10.3390/foods12203729/s1, Table S1: Volatile flavor substances and content in the inner and surface CQTB.

## References

[1] Wang X., Wang S., Zhao H. (2019). Unraveling microbial community diversity and succession of Chinese Sichuan sausages during spontaneous fermentation by high-throughput sequencing. J. Food Sci. Technol..

[2] Song Z., Cao Y., Zhang Y., Zhang Z., Shi X., Zhang W., Wen P. (2022). Effects of storage methods on the microbial community and quality of Sichuan smoked bacon. LWT.

[3] Wang Y., Wang Z., Han Q., Xie Y., Zhou H., Zhou K., Li X., Xu B. (2022). Comprehensive insights into the evolution of microbiological and metabolic characteristics of the fat portion during the processing of traditional Chinese bacon. Food Res. Int..

[4] Li X., Nie Q., Liu D., Xu Y. (2021). Changes in volatile organic compounds and lipid oxidation in traditional Chinese bacon during cold smoking. Int. J. Food Eng..

[5] Xi L., Zhang J., Wu R., Wang T., Ding W. (2021). Characterization of the Volatile Compounds of Zhenba Bacon at Different Process Stages Using GC–MS and GC–IMS. Foods.

[6] Wu H., He Z., Yang L., Li H. (2023). Volatile compounds comparison and mechanism exploration of non-smoked traditional Chinese bacon in Southwestern China and Eastern China. Food Res. Int..

[7] Xi L., Sun Y., Jiang S., Wen C., Ding W. (2023). Evaluation of effects of ultrasound-assisted curing on the flavor of Chinese bacon. Ultrason. Sonochem..

[8] Li J., Dadmohammadi Y., Abbaspourrad A. (2023). Understanding animal-based flavor generation, mechanisms and characterization: Cheddar cheese and bacon flavors. Crit. Rev. Food Sci. Nutr..

[9] Yang L., Li H., Wu H., Su C., He Z. (2023). Quality relationship between smoked and air-dried bacon of Sichuan-Chongqing in China: Free amino acids, volatile compounds, and microbial diversity. Food Res. Int..

[10] Bernardi A.O., Garcia M.V., Copetti M.V. (2019). Food industry spoilage fungi control through facility sanitization. Curr. Opin. Food Sci..

[11] Asefa D.T., Møretrø T., Gjerde R.O., Langsrud S., Kure C.F., Sidhu M.S., Nesbakken T., Skaar I. (2009). Yeast diversity and dynamics in the production processes of Norwegian dry-cured meat products. Int. J. Food Microbiol..

[12] Ashaolu T.J., Khalifa I., Mesak M.A., Lorenzo J.M., Farag M.A. (2021). A comprehensive review of the role of microorganisms on texture change, flavor and biogenic amines formation in fermented meat with their action mechanisms and safety. Crit. Rev. Food Sci. Nutr..

[13] Zhang M., Qiao H., Zhang W., Zhang Z., Wen P., Zhu Y. (2021). Tissue Type: A Crucial Factor Influencing the Fungal Diversity and Communities in Sichuan Pork Bacon. Front. Microbiol..

[14] Wang S., Wang X., Pan W., Liu A., Liu S., Yang Y., Zou L. (2021). Evaluation of Bacterial Diversity and Quality Features of Traditional Sichuan Bacon from Different Geographical Region. Appl. Sci..

[15] Mao Y., Yun J., Zhao F., Ai D., Zhang W., He K., Wang R., Wu S. (2022). Effect of Second Inoculation Time on the Volatile Flavor of Fermented Dry-Cured Meat. Food Sci..

[16] Jin G., Zhang J., Yu X., Zhang Y., Lei Y., Wang J. (2010). Lipolysis and lipid oxidation in bacon during curing and drying-ripening. Food Chem..

[17] Wang X., Zhang Y., Ren H., Zhan Y. (2017). Comparison of bacterial diversity profiles and microbial safety assessment of salami, Chinese dry-cured sausage and Chinese smoked-cured sausage by high-throughput sequencing. LWT.

[18] Guo X., Huang F., Zhang H., Zhang C., Hu H., Chen W. (2016). Classification of traditional Chinese pork bacon based on physicochemical properties and chemometric techniques. Meat Sci..

[19] Guo X., Wang Y., Lu S., Wang J., Fu H., Gu B., Lyu B., Wang Q. (2021). Changes in proteolysis, protein oxidation, flavor, color and texture of dry-cured mutton ham during storage. LWT.

[20] Chen H., Kang X., Wang X., Chen X., Nie X., Xiang L., Liu D., Zhao Z. (2023). Potential Correlation between Microbial Diversity and Volatile Flavor Substances in a Novel Chinese-Style Sausage during Storage. Foods.

[21] Du H., Chen Q., Liu Q., Wang Y., Kong B. (2021). Evaluation of flavor characteristics of bacon smoked with different woodchips by HS-SPME-GC-MS combined with an electronic tongue and electronic nose. Meat Sci..

[22] Ahn D.U., Mendonça A.F., Feng X., Toldrá F. (2017). The storage and preservation of meat: II—Nonthermal technologies. Lawrie’s Meat Science.

[23] Ramírez R., Cava R. (2007). Volatile profiles of dry-cured meat products from three different Iberian x Duroc genotypes. J. Agric. Food Chem..

[24] Marušić N., Vidaček S., Janči T., Petrak T., Medić H. (2014). Determination of volatile compounds and quality parameters of traditional Istrian dry-cured ham. Meat Sci..

[25] Liu P., Wang S., Zhang H., Wang H., Kong B. (2019). Influence of glycated nitrosohaemoglobin prepared from porcine blood cell on physicochemical properties, microbial growth and flavour formation of Harbin dry sausages. Meat Sci..

[26] Huang L., Sun Z., Zeng X., Pan D., He J., Shen J. (2021). Effects of Multi-Ingredients for Nitrite on the Volatile Flavor Compounds in Cured Meat. Zhongguo Shipin Xuebao.

[27] Panseri S., Soncin S., Chiesa L.M., Biondi P.A. (2011). A headspace solid-phase microextraction gas-chromatographic mass-spectrometric method (HS-SPME–GC/MS) to quantify hexanal in butter during storage as marker of lipid oxidation. Food Chem..

[28] Song S., Tang Q., Fan L., Xu X., Song Z., Hayat K., Feng T., Wang Y. (2017). Identification of pork flavour precursors from enzyme-treated lard using Maillard model system assessed by GC–MS and partial least squares regression. Meat Sci..

[29] Gabler F.M., Mercier J., Jiménez J.I., Smilanick J.L. (2010). Integration of continuous biofumigation with Muscodor albus with pre-cooling fumigation with ozone or sulfur dioxide to control postharvest gray mold of table grapes. Postharvest Biol. Technol..

[30] Monforte A.R., Martins S.I.F.S., Silva Ferreira A.C. (2018). Strecker Aldehyde Formation in Wine: New Insights into the Role of Gallic Acid, Glucose, and Metals in Phenylacetaldehyde Formation. J. Agric. Food Chem..

[31] Zhu W., Luan H., Bu Y., Li X., Li J., Ji G. (2019). Flavor characteristics of shrimp sauces with different fermentation and storage time. LWT.

[32] Hu Y., Zhang L., Liu Q., Wang Y., Chen Q., Kong B. (2020). The potential correlation between bacterial diversity and the characteristic volatile flavour of traditional dry sausages from Northeast China. Food Microbiol..

[33] Yin X., Wen R., Sun F., Wang Y., Kong B., Chen Q. (2021). Collaborative analysis on differences in volatile compounds of Harbin red sausages smoked with different types of woodchips based on gas chromatography–mass spectrometry combined with electronic nose. LWT.

[34] Issenberg P., Lustre A.O. (1970). Phenolic components of smoked meat products. J. Agric. Food Chem..

[35] Hu Y., Chen Q., Wen R., Wang Y., Qin L., Kong B. (2019). Quality characteristics and flavor profile of Harbin dry sausages inoculated with lactic acid bacteria and *Staphylococcus xylosus*. LWT.

[36] Chen Q., Hu Y., Wen R., Wang Y., Qin L., Kong B. (2021). Characterisation of the flavour profile of dry fermented sausages with different NaCl substitutes using HS-SPME-GC-MS combined with electronic nose and electronic tongue. Meat Sci..

[37] Saldaña E., Saldarriaga L., Cabrera J., Siche R., Behrens J.H., Selani M.M., de Almeida M.A., Silva L.D., Silva Pinto J.S., Contreras-Castillo C.J. (2019). Relationship between volatile compounds and consumer-based sensory characteristics of bacon smoked with different Brazilian woods. Food Res. Int..

[38] Pu D., Zhang Y., Zhang H., Sun B., Ren F., Chen H., Tang Y. (2020). Characterization of the Key Aroma Compounds in Traditional Hunan Smoke-Cured Pork Leg (Larou, THSL) by Aroma Extract Dilution Analysis (AEDA), Odor Activity Value (OAV), and Sensory Evaluation Experiments. Foods.

[39] Shi J., Nian Y., Da D., Xu X., Zhou G., Zhao D., Li C. (2020). Characterization of flavor volatile compounds in sauce spareribs by gas chromatography–mass spectrometry and electronic nose. LWT.

[40] Liu D., Zhao Z., Wu J., Zou Y., Wang X., Li M. (2019). Effects of Different Smoking Materials on Volatile Flavor Compounds in Smoked Chicken Thighs. Food Sci..

[41] Varlet V., Knockaert C., Prost C., Sérot T. (2006). Comparison of odor-active volatile compounds of fresh and smoked salmon. J. Agric. Food Chem..

[42] Wu W., Zhou Y., Wang G., Zhu R., Ge C., Liao G. (2020). Changes in the physicochemical properties and volatile flavor compounds of dry-cured Chinese Laowo ham during processing. J. Food Process. Preserv..

[43] Berwal J.S., Dincho D. (1995). Molds as Protective Cultures for Raw Dry Sausages. J. Food Prot..

[44] Zhou H., Zhao B., Zhang S., Wu Q., Zhu N., Li S., Pan X., Wang S., Qiao X. (2021). Development of volatiles and odor-active compounds in Chinese dry sausage at different stages of process and storage. Food Sci. Hum. Wellness.

[45] van Gemert L.J. (2011). Odour Thresholds: Compilations of Odour Threshold Values in Air, Water and Other Media.

[46] Xu M., Ye L., Wang J., Wei Z., Cheng S. (2017). Quality tracing of peanuts using an array of metal-oxide based gas sensors combined with chemometrics methods. Postharvest Biol. Technol..

[47] Sánchez Mainar M., Stavropoulou D.A., Leroy F. (2017). Exploring the metabolic heterogeneity of coagulase-negative *staphylococci* to improve the quality and safety of fermented meats: A review. Int. J. Food Microbiol..

[48] Yi L., Su G., Hu G., Peng Q. (2017). Diversity study of microbial community in bacon using metagenomic analysis. J. Food Saf..

[49] Magistà D., Susca A., Ferrara M., Logrieco A.F., Perrone G. (2017). *Penicillium* species: Crossroad between quality and safety of cured meat production. Curr. Opin. Food Sci..

[50] Ludemann V., Pose G., Pollio M.L., Segura J. (2004). Determination of growth characteristics and lipolytic and proteolytic activities of *Penicillium* strains isolated from Argentinean salami. Int. J. Food Microbiol..

[51] Khan M.I., Jo C., Tariq M.R. (2015). Meat flavor precursors and factors influencing flavor precursors—A systematic review. Meat Sci..

[52] Zhu C., Tian W., Sun L., Liu Y., Li M., Zhao G. (2019). Characterization of protein changes and development of flavor components induced by thermal modulation during the cooking of chicken meat. J. Food Process. Preserv..

[53] Petrova I., Aasen I.M., Rustad T., Eikevik T.M. (2015). Manufacture of dry-cured ham: A review. Part 1. Biochemical changes during the technological process. Eur. Food Res. Technol..

[54] Zhong A., Chen W., Hu L., Wu Z., Xiao Y., Li K., Li Z., Wang Y., Wang C. (2022). Characterisation of key volatile compounds in fermented sour meat after fungi growth inhibition. LWT.

[55] Zhang B.-Q., Luan Y., Duan C.-Q., Yan G.-L. (2018). Use of *Torulaspora delbrueckii* Co-fermentation with Two *Saccharomyces cerevisiae* Strains with Different Aromatic Characteristic to Improve the Diversity of Red Wine Aroma Profile. Front. Microbiol..

[56] Xu X., Lu S., Li X., Bai F., Wang J., Zhou X., Gao R., Zeng M., Zhao Y. (2022). Effects of microbial diversity and phospholipids on flavor profile of caviar from hybrid sturgeon (*Huso dauricus* × *Acipenser schrencki*). Food Chem..

[57] Jiang L., Chen Y., Deng L., Liu F., Wang T., Shi X., Wang B. (2022). Bacterial community diversity and its potential contributions to the flavor components of traditional smoked horsemeat sausage in Xinjiang, China. Front. Microbiol..

