# Supplementary material for: The Volatile Flavor Substances, Microbial Diversity, and Their Potential Correlations of Inner and Surface Areas within Chinese Qingcheng Mountain Traditional Bacon
Source: Foods. 2023 Oct 11;12(20):3729. doi: 10.3390/foods12203729 (PMC10606684; doi:10.3390/foods12203729)
Supplement: Supplementary file 1 [file foods-12-03729-s001.zip › foods-2601271-supplementary.pdf]

Table S1. Volatile flavor substances and content in the inner and surface CQTB.

No	Compound name and classification	CAS	Absolute content（μg/kg ）	
			Inner	Surface
	Phenols			
A1	2,5-Dimethylphenol	95-87-4	-	94.19±11.6[a]
A2	2,6-Dimethoxyphenol	91-10-1	35.15±3.59[b]	90.95±10.33[a]
A3	2-Methoxy-5-methylphenol	1195-09-1	120.04±14.46[b]	246.63±30.21[a]
A4	3,5-Dimethylphenol	108-68-9	46.19±1.26[a]	48.75±5.36[a]
A5	3-Ethyl-5-methylphenol	698-71-5	-	10.98±1.21[a]
A6	4-Ethyl-2-methoxyphenol	2785-89-9	62.08±1.60[b]	161.83±20.36[a]
A7	4-Ethylphenol	123-07-9	13.04±0.54[b]	44.04±5.12[a]
A8	2-Methoxy-4-propylphenol	2785-87-7	2.46±0.24[b]	26.49±3.12[a]
A9	Eugenol	97-53-0	2.77±0.24[b]	11.29±1.28[a]
A10	Guaiacol	90-05-1	161.01±14.57[b]	244.33±14.57[a]
A11	O-cresol	95-48-7	110.53±2.89[b]	248.09±28.56[a]
A12	P-cresol	106-44-5	92.06±8.53[b]	310.71±42.51[a]
	Aldehydes			
B1	1-Nonanal	124-19-6	168.82±12.25[b]	227.73±19.77[a]
B2	2-Ethyl-2-hexenal	645-62-5	-	37.94±4.25[a]
B3	6-(Acetoxy)-4-methyl-4-hexenal	35334-60-2	-	42.12±5.36[a]
B4	Heptanal	111-71-7	40.54±1.53[a]	31.06±4.16[b]
B5	N-hexanal	66-25-1	184.1±6.01[a]	-
B6	Phenylacetaldehyde	122-78-1	-	50.96±5.12[a]
	Hydrocarbons			
C1	2-Methylnaphthalene	91-57-6	-	12.64±1.26[a]
C2	Acenaphthene	83-32-9	3.25±0.39[b]	39.63±5.02[a]
C3	Dodecane	112-40-3	-	22.56±2.26[a]
C4	N-hexadecane	2801-87-8	1.00±0.17[a]	-
C5	N-octane	111-65-9	15.91±1.39[b]	123.54±18.3[a]

C6	α-Cedrene	469-61-4	0.82±0.14[b]	32.51±3.22[a]
	Ketones			
D1	1-(2-Hydroxy-5-methylphenyl)ethanone	1450-72-2	6.19±0.58[b]	19.11±2.25[a]
D2	1-Indanone	83-33-0	7.61±0.72[b]	16.5±1.78[a]
D3	2-Nonanone	821-55-6	-	55.69±5.65[a]
D4	Ethyl cyclopentaenol ketone	21835-01-8	14.88±1.86[b]	18.79±2.18[a]
D5	3-Methyl-2-cyclopenten-1-one	2758-18-1	-	30.8±4.12[a]
D6	Methyl cyclopentenolone	80-71-7	107.64±2.51[b]	121.17±15.21[a]
	Esters			
E1	Ethyl 2-methylbutyrate	7452-79-1	3.32±0.46[a]	-
E2	Ethyl isovalerate	108-64-5	17.95±0.61[a]	6.28±0.61[b]
	Acids			
F1	2-Methyl butyric acid	116-53-0	43.54±4.12[b]	68.09±6.12[a]
F2	Octanoic acid	124-07-2	14.61±1.83[a]	-
	Alcohols			
G1	1-Octene-3-ol	3391-86-4	-	9.82±1.20[a]
	Ethers			
H1	1,2-Dimethoxybenzene	91-16-7	28.6±2.33[b]	63.94±7.21[a]
	Others			
I1	3,4-Dimethoxytoluene	494-99-5	18.03±1.59[b]	120.82±10.25[a]
I2	1,2,3-Trimethoxybenzene	634-36-6	9.15±1.25[b]	13.92±1.49[a]
I3	1,2-Dimethoxy-4-ethylbenzene	30405-75-5	8.84±1.03[b]	35.6±4.28[a]

Different superscript letters in the same row indicate significant differences (p<0.05), CAS: Chemical Abstract Services registry number.
